# A germ cell‐specific ageing pattern in otherwise healthy men

**DOI:** 10.1111/acel.13242

**Published:** 2020-09-20

**Authors:** Sandra Laurentino, Jann‐Frederik Cremers, Bernhard Horsthemke, Frank Tüttelmann, Karen Czeloth, Michael Zitzmann, Eva Pohl, Sven Rahmann, Christopher Schröder, Sven Berres, Klaus Redmann, Claudia Krallmann, Stefan Schlatt, Sabine Kliesch, Jörg Gromoll

**Affiliations:** ^1^ Centre of Reproductive Medicine and Andrology University of Münster Münster Germany; ^2^ Institute of Human Genetics University of Duisburg‐Essen University Hospital Essen Essen Germany; ^3^ Institute of Reproductive Genetics University of Münster Münster Germany; ^4^ Institute of Human Genetics University of Münster Münster Germany; ^5^ Genome Informatics University of Duisburg‐Essen University Hospital Essen Essen Germany

**Keywords:** DNA integrity, DNA methylation, male reproductive ageing, paternal age effects, sperm

## Abstract

Life‐long sperm production leads to the assumption that male fecundity remains unchanged throughout life. However, recently it was shown that paternal age has profound consequences for male fertility and offspring health. Paternal age effects are caused by an accumulation of germ cell mutations over time, causing severe congenital diseases. Apart from these well‐described cases, molecular patterns of ageing in germ cells and their impact on DNA integrity have not been studied in detail. In this study, we aimed to assess the effects of ‘pure’ ageing on male reproductive health and germ cell quality. We assembled a cohort of 198 healthy men (18–84 years) for which end points such as semen and hormone profiles, sexual health and well‐being, and sperm DNA parameters were evaluated. Sperm production and hormonal profiles were maintained at physiological levels over a period of six decades. In contrast, we identified a germ cell‐specific ageing pattern characterized by a steady increase of telomere length in sperm and a sharp increase in sperm DNA instability, particularly after the sixth decade. Importantly, we found sperm DNA methylation changes in 236 regions, mostly nearby genes associated with neuronal development. By in silico analysis, we found that 10 of these regions are located in loci which can potentially escape the first wave of genome‐wide demethylation after fertilization. In conclusion, human male germ cells present a unique germline‐specific ageing process, which likely results in diminished fecundity in elderly men and poorer health prognosis for their offspring.

## Graphical abstract

In this study we found that healthy ageing in men is associated with a maintenance of normal sperm parameters and hormonal profiles, and maintenance of quality of life. In germ cells, a specific pattern of ageing is found, including increased telomere length, decreased DNA stability, and genome‐wide changes in DNA methylation. These changes to the germ cell genome likely result in diminished fecundity in elderly fathers.

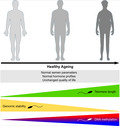

## 1 INTRODUCTION

There is a current trend to delay parenthood, due to sociopolitical and cultural reasons (Khandwala et al., 2017; Mills et al., 2011; Prioux, 2005). The reproductive implications for delayed fatherhood have been consistently undervalued due to the life‐long production of sperm. Nevertheless, the effects of ageing on male fertility are obvious, albeit often disregarded.

The consequences of advanced paternal age only recently started to become apparent. Paternal age effect (PAE) disorders (Kovac et al., 2013) are classically viewed as a consequence of the increasing number of pre‐meiotic cell divisions in the human male germline over age, leading to an increase in the *de novo* mutation rates in children of older men (Kong et al., 2012; Maretty et al., 2017). Nevertheless, mutations alone cannot explain the prevalence of PAE, and other hypotheses, such as accumulation of DNA methylation changes (Jenkins et al., 2014, 2018; Milekic et al., 2015), have been put forward to explain the detrimental effects of age on offspring health. Sperm DNA methylation has previously been shown to be influenced by fertility and lifestyle factors (Donkin & Barres, 2018; Laurentino et al., 2016). Because previous studies on age‐related changes to human sperm DNA have been performed on unselected men attending fertility clinics, it remains unclear whether these alterations might have been caused by factors other than ageing itself.

Besides PAE, some studies have associated male ageing with a decline in sperm parameters and disruptions in reproductive hormone secretion (Eskenazi et al., 2003; Feldman et al., 2002). In contrast, others have shown only subtle changes in classical reproductive parameters with age (Kelsey et al., 2014; Sartorius & Nieschlag, 2010). These discrepancies might have been due to variable selection criteria for clinical studies, since unhealthy lifestyles and age‐related somatic diseases can have a strong impact on fertility and hormonal profiles (Zitzmann, 2013).

Observational studies have shown that partners of elderly men take longer to become pregnant and show increased risk for miscarriage (Koh et al., 2013; Mutsaerts et al., 2012; Rochebrochard & Thonneau, 2002). Moreover, advanced paternal age has been shown to have adverse consequences for maternal and offspring health (Khandwala et al., 2018). However, similar to semen parameters and hormonal profiles in elderly men, it is unclear whether these effects are caused by the ageing process itself or by co‐morbidities.

With the steadily increasing trend for delaying fatherhood, the impact of advanced paternal age (i.e. increase in ART procedures, poorer perinatal health) might influence the health of the upcoming generations. Therefore, it is important to identify the reproductive parameters which are affected by age itself and differentiate them from those impacted by life style or other co‐morbidities.

By studying a cohort of healthy men, we identified age‐dependent changes in the male germline which are not caused by age‐associated diseases, but can be exclusively attributed to ageing.

## 2 RESULTS

### 2.1 Reproductive parameters in healthy ageing men

We have assembled a cross‐sectional observational cohort of healthy men (FAMe, Fertility and Ageing in Healthy Men) from which we collected clinical data and analysed several molecular features of ageing in sperm. Reproductive parameters were evaluated in 197 healthy men (Table S1). No age‐related changes in testicular volume or semen parameters relative to the normal ranges given by the WHO were observed in our cohort (World Health Organization, 2010). Subtle but significant changes, such as a decrease in ejaculate volume, changes in sperm motility, and a decrease in markers for accessory gland function could be observed, though they remained within normal physiological ranges (Table 1 and Table S2). Concerning reproductive endocrine parameters, we noticed a slight, but significant age‐dependent increase in follicle‐stimulating hormone (FSH) and sex hormone binding globulin (SHBG), and a decrease in free testosterone; however, again all hormone levels stayed within the normal range (Table 1). A similar scenario was observed when evaluating anthropometric parameters and questionnaires for sexual health and well‐being (Table S3).

**TABLE 1:** Main andrological parameters across age groups

	Group 1 n = 34 18–25 years	Group 2 n = 36 26–35 years	Group 3 n = 28 36–45 years	Group 4 n = 39 46–55 years	Group 5 n = 36 56–65 years	Group 6 n = 24 >66 years	Correlation with age
Testicular volume (>15 cm^3^)	43.2 (13.4)	41.5 (13.8)	42.3 (15.7)	39.1 (12.5)	42.7 (12.9)	39.6 (13.4)	−0.05 (0.51)
Ejaculate volume (≥1.5 ml)	4.2 (1.7)	4.6 (2.3)	3.7 (1.8)	3.2 (1.5)	2.9 (1.5)	2.0 (1.2)	−0.41 (3.86 × 10^−9^
Sperm concentration (≥15 million/ml)	39.5 (37.3)	47.6 (37.8)	34.5 (30.7)	53.5 (45.1)	52.7 (50.1)	62.9 (43.8)	0.08 (0.29)
Total sperm count (≥39 million)	142.3 (116.2)	206.5 (179.5)	118.7 (86.0)	165.4 (160.3)	139.1 (140.5)	122.3 (120.8)	−0.14 (0.06)
Progressive motility (≥32%)	55.2 (15.1)	57.9 (12.5)	47.8 (16.4)	49.8 (16.2)	43.4 (18.7)	35.6 (21.1)	−0.39 (1.68 × 10^−8^)
Normal morphology (≥4%)	4.9 (1.7)	4.7 (1.8)	4.6 (2.0)	5.2 (2.1)	4.4 (2.2)	5.1 (2.1)	0.01 (0.85)
pH (≥7.2)	8.1 (0.3)	8.1 (0.3)	8.3 (0.3)	8.2 (0.3)	8.4 (0.5)	8.2 (0.3)	0.19 (0.009)
Vitality (>58%)	71.2 (11.5)	70.5 (11.4)	66.0 (14.2)	69.3 (11.4)	60.6 (15.8)	59.3 (15.9)	−0.31 (1.99 × 10^−5^)
FSH (2–10 U/l)	3.0 (1.9)	3.4 (1.5)	4.0 (2.0)	4.1 (2.4)	5.2 (5.9)	6.1 (3.0)	0.36 (2.08 × 10^−7^)
LH (1–7 U/l)	3.0 (0.9)	3.2 (1.1)	3.5 (1.5)	3.0 (1.1)	2.8 (1.2)	4.0 (1.7)	0.02 (0.81)
Total testosterone (>12 nmol/l)	23.9 (7.3)	23.8 (6.7)	21.2 (6.8)	19.4 (6.2)	20.8 (6.2)	24.1 (9.3)	−0.12 (0.09)
SHBG (11–71 nmol/l)	38.2 (12.8)	36.6 (12.8)	37.2 (11.9)	39.7 (13.1)	47.3 (17.7)	58.8 (19.3)	0.32 (3.56 × 10^−6^)
Free testosterone (>250 pmol/l)	494.4 (128.5)	501.5 (103.8)	435.8 (138.7)	369.0 (111.3)	361.2 (84.9)	361.3 (117.4)	−0.47 (6.89 × 10^−12^)
DHT (0.5–2.0 nmol/l)	0.9 (0.2)	0.8 (0.2)	0.8 (0.2)	0.8 (0.3)	0.8 (0.3)	1.0 (0.4)	−0.004 (0.95)
Oestradiol (<250 pmol/l)	89.0 (31.3)	86.8 (26.9)	85.6 (34.8)	78.1 (28.8)	88.5 (32.2)	98.1 (33.4746)	0.01 (0.87)
Prolactin (<500 mU/l)	297.0 (165.0)	303.0 (204.4)	225.5 (97.2)	189.4 (118.2)	165.3 (92.6)	199.4 (56.9)	−0.38 (3.43 × 10^−8^)
Prostate‐specific antigen (<4 µg/l)	0.5 (0.2)	0.6 (0.3)	0.7 (0.5)	0.9 (0.8)	1.5 (1.3)	1.9 (1.7)	0.45 (2.70 × 10^−11^)

Note

Normal ranges are indicated for each parameter. Results are shown as mean (*SD*) for each age group and Spearman's rank correlations with age as *ρ* (*p*‐value).

Next, we studied three hallmarks of somatic cell ageing namely telomere attrition, DNA integrity and DNA methylation changes.

### 2.2 Sperm telomere length increases with age

**FIGURE 1:**
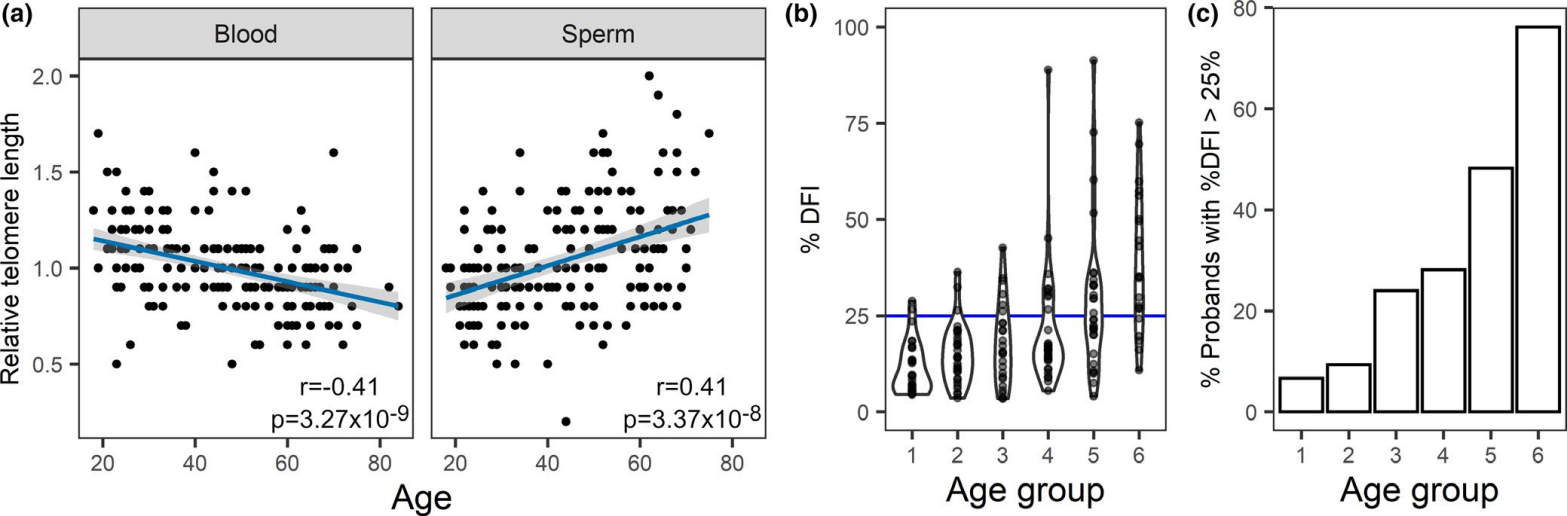
(a) Relative telomere length was determined in peripheral blood (n = 194) and swim‐up (n = 179) sperm DNA. Linear trends are displayed in blue and 95% confidence intervals in grey shading. The Pearson correlation coefficients and p‐value are indicated for each case. (b and c) Sperm DNA fragmentation index increases with age. (a) Distribution of DFI values across the age groups is shown in the form of a violin plot. The normal upper value of 25% is marked in blue. (b) The percentage of men in each age group with pathological levels of DFI increases with age

We found an inverse association between age and relative telomere length in peripheral blood cells (rTL; Figure 1a), indicating the age‐dependant shortening of telomeres in somatic cells (slope = −0.0055). In contrast, there was a significant positive association of similar strength between age and sperm rTL, indicating lengthening of germline telomeres with increasing age (slope = 0.0077).

### 2.3 Sperm DNA instability increases with age

Investigating genomic instability, a somatic hallmark of ageing, we set out to evaluate how the stability of the human sperm genome varies with progressive age. To do so, we measured sperm DNA fragmentation, which quantifies single and double strand breaks in sperm DNA (Evenson, 2016). We observed a highly significant increase in DFI with increasing age (*ρ* = 0.57; *p* = 1.04 × 10^−15^; Figure 1b). Possible confounding factors, such as abstinence time and sperm vitality, had little to no effect on this association (Table S4). A level of 25% DFI is usually used in the clinics as the maximum normal value of DNA fragmentation (Evenson, 2016). The number of men with DFI values above this threshold increases with age, especially in the two oldest age groups, with almost 80% of men over the age of 66 showing pathological DFI levels (Figure 1c).

### 2.4 Sperm DNA shows widespread DNA methylation changes with age

Another hallmark of ageing seen in somatic cells is the commonly designated epigenetic drift, in which DNA methylation changes occur during ageing. In order to explore age‐dependent methylation changes in sperm in an unbiased and genome‐wide manner, we performed whole genome bisulphite sequencing on sperm DNA from six of the youngest and oldest probands in our study (18–25 years vs. older than 65 years). For comparison, blood DNA from the same men was prepared and sequenced. There were no differences in mean DNA methylation between age groups or between tissues (Table S5), and absence of somatic contamination was confirmed by evaluating germline‐specific promoters (examples shown on Figure S1).

Differentially methylated regions (DMRs; with at least 4 CpGs and 30% mean methylation difference) between the two groups were identified using two different algorithms (BSmooth and Metilene). We identified a total of 236 DMRs in sperm (File S1). Of the sperm DMRs, 121 show increased and 115 decreased methylation with age. We did not find any chromosomal preference or hotspot for the observed sperm methylation changes (Figure S2). Notably, there was no overlap between the age‐associated sperm DMRs and blood DMRs. Only sperm DMRs were analysed further as somatic ageing falls outside the scope of our work.

**FIGURE 2:**
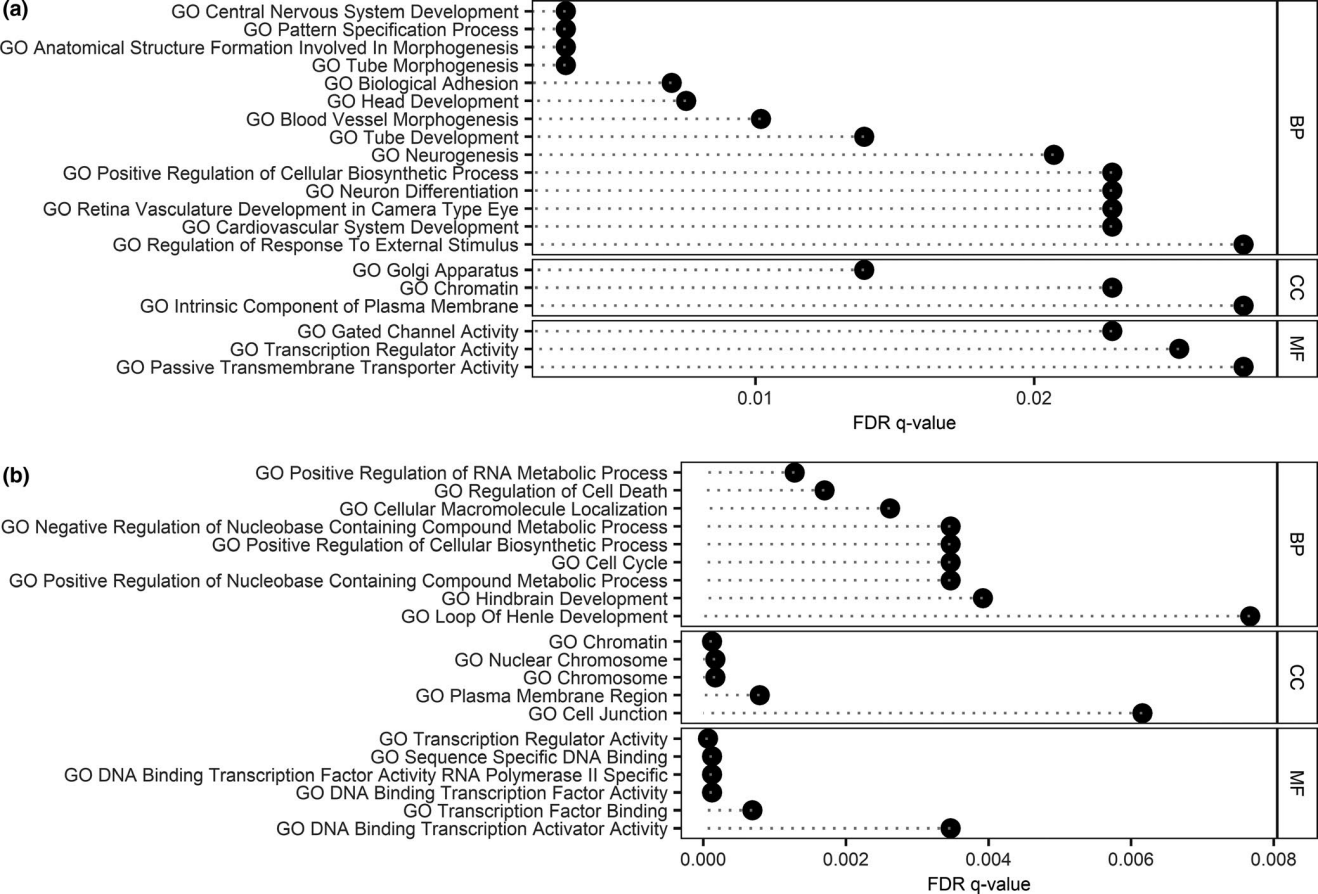
Biological impact of identified hyper‐ (a) and hypomethylated (b) DMRs. Overlap of the DMR‐associated gene list with gene ontology (GO) data sets is shown along with the false discovery rate (FDR) *q*‐value. The 20 pathways with lowest FDR in the GO domains biological process (BP), cellular component (CC) and molecular function (MF) are displayed

There was a significant over‐representation of DNA transposons (*p* = 9.59 × 10^−4^) and LINE elements (*p* = 6.21 × 10^−3^) in the identified sperm DMRs, but no significant over‐ or under‐representation of either LTRs or SINE (Figure S3). Gene ontology (Huang et al., 2009) analysis of the up‐ and downstream neighbouring genes (≤1000 kb from TSS) revealed a significant enrichment for homeobox genes (11 genes; *p*_adj_ = 0.02), DNA binding (35 genes; *p*_adj_ = 0.009), nucleus (66 genes, *p*_adj_ = 0.02) and transcription (36 genes; *p*_adj_ = 0.04) for the hypomethylated regions. For the hypermethylated regions, none of the enrichments reached significance after Benjamini–Hochberg multiple testing correction. Gene set enrichment analysis (GSEA) (Liberzon et al., 2011; Subramanian et al., 2005) showed significant enrichment in several gene sets (FDR < 0.05) for both the hyper‐ and hypomethylated DMR‐associated gene sets. The 20 gene ontology (GO) terms with lowest FDR (Figure 2) included terms associated with transcriptional regulation (e.g. transcription regulator activity, sequence specific DNA binding, transcription factor binding) and with nervous system development (e.g. central nervous system development, head development, neurogenesis).

### 2.5 Do the identified germ cell DMRs escape genome‐wide demethylation?

To address to which extent the identified age‐related changes in sperm DNA methylation have any impact on fertilization, development or even offspring health, we compared the location of the detected DMRs with regions that escape genome‐wide demethylation after fertilization. For this purpose, we have used publicly available single cell WGBS data from human gametes and embryos (Zhu et al., 2018) to track the methylation dynamics of the age‐associated sperm DMRs. We considered genomic regions with persistent DNA methylation above 30% (as published by Tang et al., 2015) and variation in DNA methylation below 30% until late pronucleus state to escape the first wave of reprogramming. This analysis revealed that 10 DMRs, designated age‐associated escapee DMRs, overlapped with reprogramming‐resistant regions. These regions included only DMRs which increased methylation with age (examples for DMRs are depicted in Figure S4; complete list of escapee DMRs in File S1).

### 2.6 DNA methylation of selected DMRs allows for sperm‐based age determination

**FIGURE 3:**
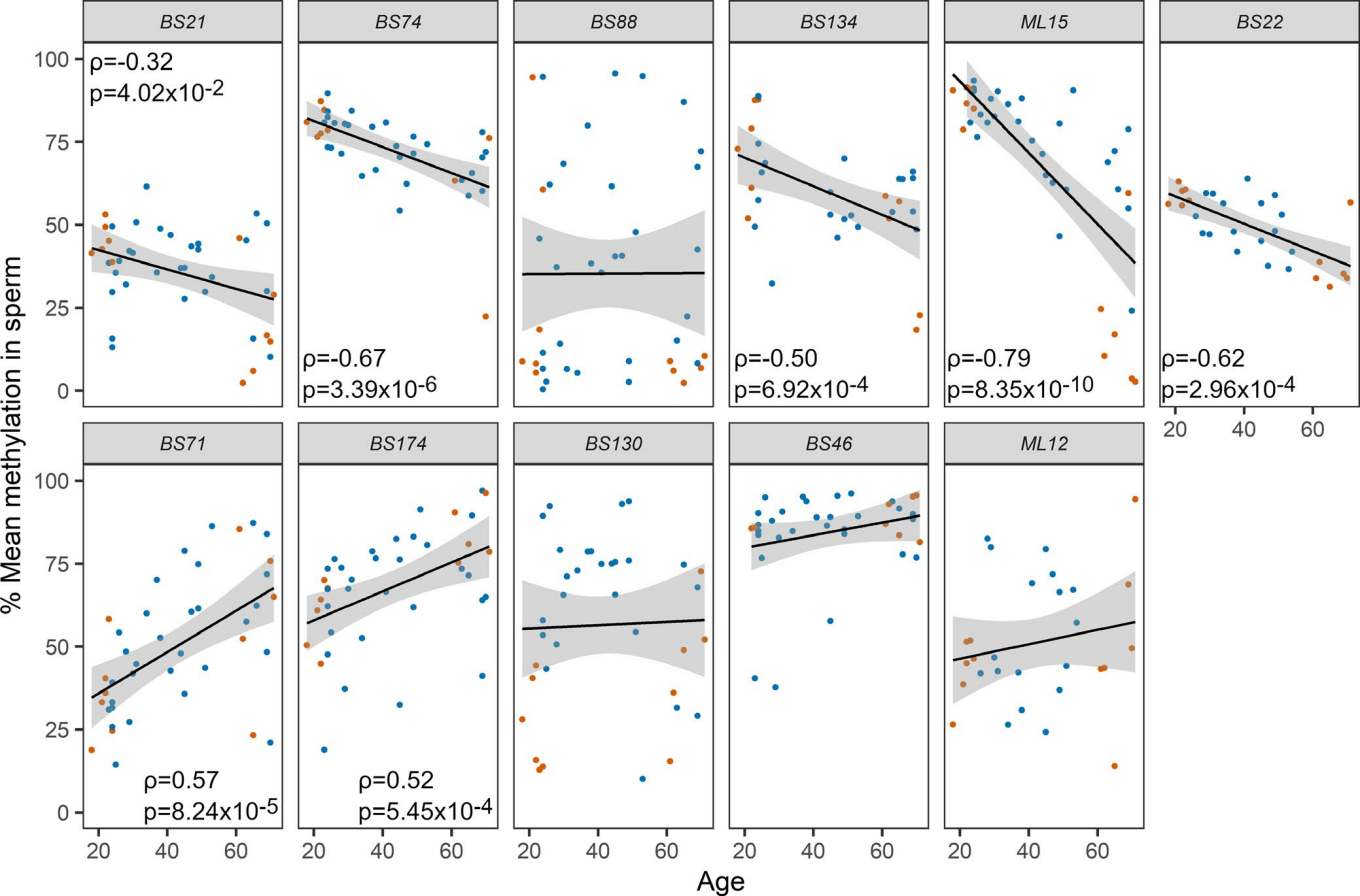
Evaluation of DMRs for age‐dependent changes by DBS. Eleven age‐associated differentially methylated regions (DMRs) identified by WGBS of sperm of young (G1) and old (G5‐6) men were further analysed in all age groups by DBS in a validation cohort (n = 42). The upper and lower panels show DMRs showing hypo‐ and hypermethylation with age, respectively. The linear trends and 95% confidence intervals are shown by solid lines and grey shading, respectively. The 12 samples previously pooled for WGBS analysis are shown in orange. A statistically significant correlation (Spearman's rank correlation) with age was detected for seven of these DMRs. Only statistically significant correlation results are displayed in each graph

In order to explore the dynamics of DNA methylation changes along the ageing process and to confirm the WGBS results, we selected a subset of 11 sperm DMRs (6 hypo‐ and 5 hypermethylated with age) from the 236 identified to be further analysed and validated using targeted deep bisulphite sequencing (DBS) analysis (Table S6). The validation cohort consisted of the 12 samples previously analysed by WGBS as pools and additional 30 randomly selected samples (DMR validation cohort; Figure 3, upper and lower panels show hypo‐ and hypermethylated DMRs, respectively). We found statistically significant correlations between DNA methylation and age for 7 of the 11 DMRs (5 hypo‐ and 2 hypermethylated). Removing the 12 samples previously analysed by WGBS as pools did not result in a change in direction of association between DNA methylation and age.

**FIGURE 4:**
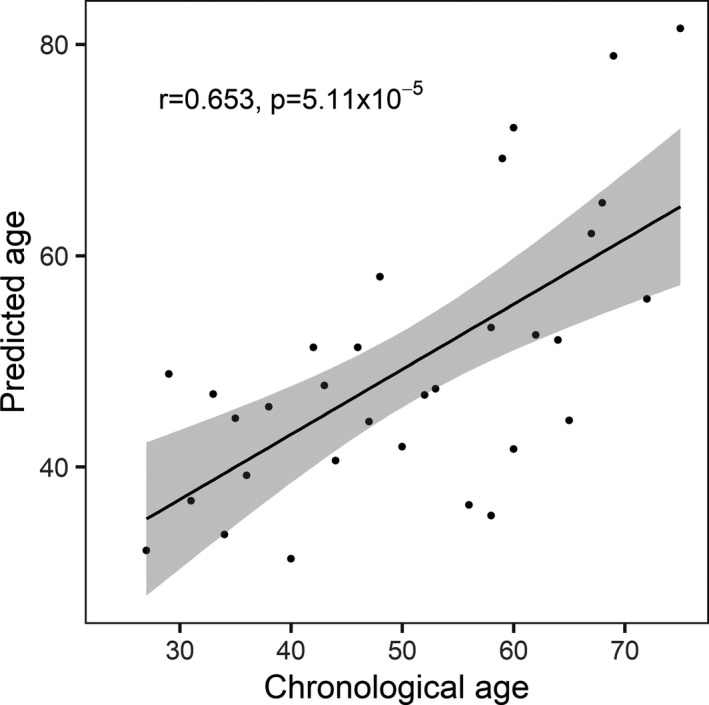
Sperm age predictor. The average methylation values of the six DMRs with a *p* < 0.001 were used for regression analysis to develop an age predictor. An independent predictor validation cohort (n = 33) was analysed, and the age predictor tool could be successfully applied to calculate the predicted age. The calculated mean absolute error including all the samples was +/− 9.8 years, with a Pearson correlation coefficient of 0.653 (*p* = 5.11 × 10^−5^)

We made use of the age‐dependent pattern of methylation and used the six DMRs with the lowest *p*‐value for the association between DNA methylation and age (BS74, BS134, ML15, BS22, BS71 and BS174) and a regression analysis of donor‐wise average methylation levels to derive an age predictor for sperm. By leave‐one‐out cross‐validation (2011), we found predicted and chronological ages for the 42 initially analysed samples to be highly correlated. Experimentally, this initial cohort showed a mean absolute error of 7.8 year in the prediction. An independent set of 33 samples was analysed for validation of the predictor, showing a moderate correlation between calculated and chronological age (Figure 4; R script available in File S2) and a mean absolute error of 9.8 years.

## 3 DISCUSSION

The FAMe study allowed to delineate for the first time an inherent ageing process taking place in the human male germline. By excluding or minimizing the effects of age‐related confounding factors, we isolated the effects caused solely by ageing. We identified a molecular germ cell‐specific ageing process (telomere lengthening, increased sperm DNA fragmentation and widespread DNA methylation changes) which might be associated with the poorer fertility outcome of elderly fathers.

### 3.1 Clinical evaluation shows healthy ageing is associated with normal reproductive parameters

All the study participants underwent an andrological evaluation which revealed that normal spermatogenesis is not affected by age in a clinically relevant manner over a range of six decades. Moreover, reproductive hormone profiles remained within physiological levels across the age range analysed. These findings are in contrast with several studies showing a decline in spermatogenic function with age (Eskenazi et al., 2003; Lee et al., 2009; Sartorius & Nieschlag, 2010). However, our findings are in perfect agreement with a previous study in which rigorous selection criteria were employed (Nieschlag et al., 1982). The enrolment criteria appear, therefore, to cause the discrepancies between different studies. Since we do not have longitudinal data, we cannot, however, exclude the influence of year of birth on the results.

An interesting observation from our study is that general health is associated with maintained fertility parameters. There is a growing body of evidence supporting the notion that male infertility, or at least aberrant sperm parameters, might be associated with poor general health (Eisenberg et al., 2015, 2016; Latif et al., 2017). In fact, some studies have also indicated that infertile men might be at a higher risk for developing cancer (Al‐Jebari et al., 2019; Hanson et al., 2016). Our study seems to add further validity to this hypothesis that male infertility/abnormal sperm production is a symptom for broader health issues by showing that abnormal sperm and hormonal parameters are less common in healthy men, regardless of age.

### 3.2 A germ cell‐specific process of molecular ageing

In addition to classical parameters for the evaluation of male fertility, we have also performed molecular analyses on sperm DNA obtained from the participants. In particular, we assessed parameters which have previously been found to change in an age‐dependent manner in somatic tissues, such as telomere length, genomic stability and DNA methylation (López‐Otín et al., 2013).

We found a positive correlation between age and sperm rTL, which is of similar strength to the negative correlation between age and blood rTL. Thus, in the germline, telomeres extend with increasing age. This phenomenon has been previously observed and attributed to telomerase activity in the germline (Ozturk, 2015). To which extent this telomere lengthening has an impact on fertility and progeny health is currently unclear. There are reports that lengthened sperm telomeres may be detrimental to fertility (Cariati et al., 2016) and could be found in the progeny of elderly fathers; how this might affect their general health is currently unclear (Eisenberg & Kuzawa, 2018).

The increased percentage of sperm DFI in elderly men, which affects almost 80% men in the oldest age group, indicates a progressive increase in genomic instability in the germline. This accelerated rate of DNA strand breaks seems to be persistent independent of the health status since it was also shown for other men for which selection criteria were not as strict as those used for the FAMe cohort (Wyrobek et al., 2006). Oxidative stress, one of the hallmarks of ageing, which has been indicated to increase in sperm with age, might be involved in the mechanism by which DNA fragmentation increases in older men (Cocuzza et al., 2008). The observed high levels of DNA fragmentation in the sperm of older men might contribute to the previously reported longer time to pregnancy and higher miscarriage rates, irrespective of female partner age (Kennedy et al., 2011; Spano et al., 2000).

By performing WGBS, we have identified 236 regions showing differences in DNA methylation between the oldest and youngest age groups. We used pools for this exploratory analysis in order to exclude interindividual variation and find common changes in sperm DNA methylation with age. This is, to our knowledge, the first study investigating age‐related methylation changes in all CpG dinucleotides in the human sperm genome. It is also the first time such changes were analysed in men selected for their health, thereby excluding the influence of lifestyle factors such as smoking or excessive alcohol consumption which themselves might alter sperm DNA methylation. Remarkably, these DMRs were sperm specific and did not overlap with age‐associated regions found in blood in this study. However, we cannot exclude that our blood methylome results might be influenced by changes in cellular composition (Jaffe & Irizarry, 2014). Nevertheless, our results, along with previous studies, strongly support the notion that epigenetic ageing in the male germline is distinct from somatic cell ageing.

The genes in the proximity of the age‐associated sperm DMRs were enriched in homeobox genes (e.g. *HOXA1* nearby BS71 and *SHOX* nearby BS174), which are essential for embryogenesis, and neuronal development (for comprehensive reviews see Mallo, 2018 and Philippidou & Dasen, 2013). To further investigate the putative functions associated with the DMR‐associated genes, we performed gene set enrichment analysis, which revealed several GO terms linked to nervous system development. Interestingly, a related finding in a group of men analysed for sperm DNA methylation changes at two time points (9–19 years apart), for which an association with genes involved in schizophrenia and bipolar disorder was observed, corroborates our analyses (Jenkins et al., 2014).

In order to have any effect on development or offspring health, changes in sperm DNA methylation must bypass the wave of genome‐wide reprogramming occurring during early development, in which most of the genome is demethylated (Lee et al., 2014). Recently, it has become clear that this phase does not erase all epigenetic marks present in the parental genomes (Tang et al., 2015; Zhu et al., 2018). These reprogramming‐resistant regions include not only repetitive elements but also some loci involved in neurological disorders (Tang et al., 2015). Indeed, using publically available data sets (Zhu et al., 2018), our bioinformatical analysis indicates that 10 out of the 236 DMRs we detected in our study might escape reprogramming. We have restricted our analysis to the Zhu data set as it was constituted by trios and the genetic background influence could be minimized. For this reason, we have not analysed whether some of these regions might also escape the (germline‐specific) second wave of demethylation.

One of the limitations of this study is that we performed WGBS analysis on pools. This has the advantage of detecting common changes occurring with age across individuals but did not allow for the detection of interindividual variation in DNA methylation. We then proceeded to validate the WGBS results in a larger number of samples using a different method, DBS. This increased our confidence in the results obtained and allowed for higher resolution analysis in a wider cohort and on an individual basis. However, due to technical limitations, this was only possible for eleven DMRs. The development of library preparation methods using lower input and the improvement in sequencing methods will allow, in the future, the analysis of the entire methylome of multiple individuals, allowing the analysis of both common and individual‐specific changes at high statistical power.

Out of eleven DMRs that underwent validation, nine DMRs showed a similar pattern as observed in WGBS, and seven showed a statistically significant correlation between age and DNA methylation in a validation cohort. Based on the six DMRs with the lowest *p*‐values for this correlation, we devised an age predictor. Very recently, a sperm epigenetic clock was published which shows higher precision than the one described here (Jenkins et al., 2018). We believe the existence of one does not invalidate the other. To build these predictors, different cohorts—unselected men attending a fertility clinic were used by Jenkins and colleagues while we recruited healthy volunteers—and DNA methylation analysis methods were used. The data necessary to predict age are therefore different (targeted sequencing for our predictor and methylation array for Jenkins and colleagues'), making them applicable in different laboratory setups, as bisulphite sequencing, especially at low resolution, is more widely available than array technology.

A crucial question that emerges from our study is: what is the origin of the DNA methylation changes in sperm? During spermiogenesis (the final step of spermatogenesis), DNA is tightly packed with protamines and therefore inaccessible to DNA methyltransferases and TET enzymes, involved in methylation and demethylation, respectively. Therefore, it is unlikely that DNA methylation changes take place at this stage. The more likely source for the age‐dependent alterations, however, is the spermatogonial stem cells. It is assumed that, in humans, each spermatogonial stem cell divides once every 16 days. That translates to around 23 divisions every year, which means that by the time a man reaches the age of 75 years, his spermatogonia are thought to have undergone nearly 1500 mitotic cell divisions (Goriely, 2016). This has long been suggested to make them prone to replication errors, resulting in increased mutation rates (Crow, 2000). It is likely that these early germ cells are also prone to increased DNA methylation maintenance errors, similar to other somatic tissues during ageing, leading to a germ cell‐specific pattern of epigenetic drift. Moreover, our group has recently described that, in order to maintain sperm production levels, testes of older men require activation and recruitment of additional, normally quiescent, spermatogonia (Pohl et al., 2019). These changes in spermatogonial activity pattern can lead to replication stress and stem cell exhaustion, well‐known hallmarks for somatic cell ageing. In addition, by changing the relative contribution of spermatogonial clones to the overall sperm output, the altered clonal composition might result in a different average DNA methylation in certain regions.

In summary, there are intrinsic ageing processes occurring in the male germline and affecting sperm DNA integrity such as telomere lengthening, increased genomic instability and changes in DNA methylation which present a unique germ cell‐specific ageing programme. This ageing pattern is present also in ageing men with good overall and reproductive health status. The different factors might negatively affect germ cell DNA to an extent which could explain the well‐known observation of reduced fertility and increased miscarriage rate of older men. It has been previously advised that genetic counselling should be provided for couples undergoing assisted reproduction when the male partner is above 49 years of age (Jennings et al., 2017; Toriello et al., 2008). Our data support this recommendation; however, further work is necessary to understand the molecular mechanisms leading to the observed changes in sperm DNA.

## 4 EXPERIMENTAL PROCEDURES

### 4.1 Ethics

All participants gave informed written consent for performing the examinations, evaluation of clinical data and genetic analysis of the DNA samples according to protocols approved by the Ethics Committee of the Medical Faculty in Münster (2013‐255‐f‐S).

Details of experimental procedures are given in File S3.

## CONFLICT OF INTEREST

The authors have no conflicts of interest.

## AUTHOR CONTRIBUTIONS

S.L. did the experimental study design, sample and data analyses, evaluated clinical parameters and wrote the manuscript. J.‐F.C. did the clinical study design, proband recruitment and data analyses. B.H. did the epigenetic study design and WGBS analyses, data evaluation and contributed to the manuscript. F.T. contributed to the experimental and clinical study design and the manuscript. K.C. worked on the study design, online questionnaire, the ethical vote and proband recruitment. M.Z. worked on the study design, proband recruitment and examination. E.P. did experimental work, was involved in data interpretation and worked on the manuscript. S.R. performed the bioinformatics analyses of the WGBS data. C.S. set up the epigenetic clock and did WGBS analyses. S.B. performed DMR and escapee analyses. K.R. did the DFI analysis. C.K. worked on the study design, proband recruitment and examination. St.S. worked on the study design, analysed the data and worked on the manuscript. S.K. was responsible for the clinical study design, the ethical permission and proband examination. J.G. developed the concept of the study, performed clinical and experimental data analyses, and wrote the manuscript.

## Data Availability

Whole genome bisulphite sequencing (WGBS) data were deposited at the European Nucleotide Archive under the accession number PRJEB28044.
