# Supplementary material for: A germ cell‐specific ageing pattern in otherwise healthy men
Source: Aging Cell. 2020 Sep 20;19(10):e13242. doi: 10.1111/acel.13242 (PMC7576283; doi:10.1111/acel.13242)
Supplement: Supplementary file 1 [file ACEL-19-e13242-s001.docx]

**Table S1** Age distribution of the FAME cohort

| Group | Age Range (years) | N |
| --- | --- | --- |
| 1 | 18-25 | 34 |
| 2 | 26-35 | 36 |
| 3 | 36-45 | 28 |
| 4 | 46-55 | 39 |
| 5 | 56-65 | 36 |
| 6 | ≥66 | 24 |

**Table S2** Additional semen parameters evaluated

|  | Group 1  n=34  18-25 years | Group 2  n=36  26-35 years | Group 3  n=28  36-45 years | Group 4  n=39  46-55 years | Group 5  n=36  56-65 years | Group 6  n= 24  >66 years | Correlation with age |
| --- | --- | --- | --- | --- | --- | --- | --- |
| Glucosidase (>20 mU/Ejac.) | 87.0 (56.4) | 131.4 (76.3) | 80.3 (46.8) | 101.3 (76.0) | 120.4 (90.9) | 113.3 (70.9) | 0.09 (0.24) |
| Fructose (>13 μmol/Ejac.) | 64.2 (28.6) | 70.5 (54.1) | 48.3 (30.3) | 39.1 (25.9) | 35.0 (30.3) | 19.2 (19.5) | -0.47 (1.18x10-11) |
| Zinc (>2.4 μmol/Ejac) | 6.8 (4.3) | 8.8 (6.4) | 7.5 (5.1) | 6.7 (3.8) | 5.8 (4.6) | 6.8 (6.0) | -0.13 (0.09) |
| Non progressive motile (%) | 12.1 (6.8) | 10.3 (3.2) | 14.5 (8.7) | 9.7 (4.4) | 9.7 (5.9) | 11.1 (7.5) | -0.07 (0.34) |
| Immotile (%) | 32.7 (10.547) | 31.9 (9.0) | 37.9 (15.9) | 40.5 (17.2) | 41.8 (17.4) | 51.6 (21.2) | 0.37 (1.05x10^-7^) |
| Head defects (%) | 95.0 (3.5) | 95.4 (2.7) | 96.0 (2.0) | 95.3 (2.1) | 96.1 (2.1) | 95.7 (2.0) | 0.05 (0.53) |
| Midpiece defects (%) | 54.4 (7.6) | 53.8 (7.4) | 54.6 (7.8) | 53.0 (6.7) | 53.0 (8.9) | 54.6 (7.0) | -0.01 (0.89) |
| Tail defects (%) | 15.6 (4.5) | 15.7 (4.9) | 15.4 (4.1) | 15.6 (5.1) | 16.2 (4.8) | 15.2 (4.3) | -0.02 (0.79) |
| Agglutination | 3.2 (7.2) | 5.8 (13.1) | 3.2 (10.1) | 3.6 (6.6) | 5.6 (13.3) | 9.1 (19.7) | 0.03 (0.69) |
| MAR IgG (<50%) | 5.9 (14.0) | 3.2 (8.3) | 8.0 (22.0) | 10.7 (23.0) | 8.2 (12.7) | 12.1 (25.3) | 0.15 (0.047) |
| MAR IgA (<50%) | 1.9 (6.2) | 1.1 (3.6) | 3.1 (8.5) | 5.7 (12.3) | 4.5 (7.8) | 6.7 (20.5) | 0.16 (0.04) |
| Round cells (mill/ml) | 1.3 (1.6) | 1.4 (1.8) | 1.2 (1.2) | 1.2 (1.3) | 1.6 (2.0) | 2.3 (3.9) | 0.05 (0.54) |
| Leucocytes (<1 mill/ml) | 0.4 (1.5) | 0.2 (0.7) | 0.2 (0.4) | 0.2 (0.4) | 0.5 (1.4) | 0.6 (1.2) | 0.05 (0.47) |

Normal ranges are indicated for each parameter. Results are shown as mean (SD) for each age group and Spearman’s rank correlations with age as ρ (p-value).

**Table S3** Anthropometric parameters and questionnaire results of the men in the cohort

|  | Group 1  n=34  18-25 years | Group 2  n=36  26-35 years | Group 3  n=28  36-45 years | Group 4  n=39  46-55 years | Group 5  n=36  56-65 years | Group 6  n= 24  >66 years | Correlation with age |
| --- | --- | --- | --- | --- | --- | --- | --- |
| BMI (18.5-<25 kg/m2) | 22.4 (2.3) | 24.3 (2.4) | 26.7 (3.6) | 26.1 (3.9) | 26.1 (3.1) | 26.9 (2.6) | 0.45 (4.00x10^-11^) |
| Waist Circumference (<102 cm) | 82.4 (1.1) | 88.6 (8.0) | 96.5 (2.6) | 97.2 (2.0) | 97.6 (10.0) | 99.4 (11.7) | 0.55 (1.02x10^-15^) |
| RR sys (90-120 mmHg) | 136.1 (23.4) | 134.8 (10.9) | 140.2 (14.0) | 148.6 (17.8) | 146.4 (14.3) | 157.5 (22.2) | 0.43 (3.27x10^-10^) |
| RR dia (60-80 mmHg) | 78.1 (15.4) | 78.8 (9.8) | 84.2 (9.5) | 92.00 (12.2) | 88.8 (8.4) | 91.6 (11.4) | 0.49 (5.84x10^-13^) |
| Prostate volume (cm^3^) | 17.2 (4.1) | 18.3 (5.1) | 18.9 (4.6) | 24.2 (7.2) | 27.6 (11.4) | 34.1 (13.7) | 0.63 (<2.2x10^-16^) |
| AMS (≤26 points) | 18.9 (2.3) | 20.0 (3.2) | 21.2 (4.0) | 24.0 (6.2) | 24.2 (5.3) | 24.7 (5.0) | 0.46 (9.03x10^-11^) |
| IIEF (26-30 points) | 29.3 (1.1) | 29.2 (1.2) | 28.5 (2.5) | 27.1 (4.0) | 25.4 (6.3) | 20.9 (6.8) | -0.48 (3.14x10^-12^) |
| IPSS (≤7 points) | 1.71 (1.5) | 3.1 (3.3) | 3.2 (2.3) | 3.8 (3.0) | 4.2 (3.3) | 6.9 (4.1) | 0.37 (2.92x10^-7^) |
| Epworth (<6 points) | 4.65 (2.8) | 4.9 (3.1) | 5.1 (2.5) | 6.00 (3.3) | 5.4 (3.9) | 5.4 (2.9) | 0.09 (0.24) |
| CAG (<24 repeats) | 21.35 (2.5) | 22.0 (3.7) | 23.3 (4.7) | 22.1 (3.1) | 22.8 (2.7) | 21.9 (2.7) | 0.08 (0.29) |

Normal ranges are indicated for each parameter. Results are shown as mean (SD) for each age group and Spearman’s rank correlations with age as ρ (p-value).

**Table S4** Adjustment of the association (by Spearman’s rank correlation) between sperm DNA fragmentation index (%DFI) and age for possible confounding factors

| Confounder | ρ (p-value) |
| --- | --- |
| None | 0.5661428 (1.044x10^-15^) |
| Abstinence time | 0.525739 (2.536657x10^-12^) |
| Sperm concentration (mill/mL) | 0.5649652 (2.304839x10^-14^) |
| Progressive motility (%) | 0.4304968 (2.508537x10^-8^) |
| Morphology (% normal forms) | 0.5368845 (7.093121x10^-13^) |
| Vitality (% live spermatozoa) | 0.4717895 (1.842531x10^-9^) |
| All of the above | 0.4305702 (8.870944x10^-8^) |

**Table S5** Whole genome bisulfite analysis efficiency and overall methylation in the analysed samples

| **Cell type** | **Mapping  efficiency** | **Duplication Rate** | **Conversion rate** | **Coverage** | **CpGs covered** | **Overall methylation** |
| --- | --- | --- | --- | --- | --- | --- |
| “Young” Sperm | 0.98 | 0.08 | 1.00 | 16.34 | 0.93 | 0.71 |
| “Old” Sperm | 0.98 | 0.13 | 1.00 | 16.32 | 0.92 | 0.72 |
| “Young” Blood | 0.98 | 0.06 | 1.00 | 19.29 | 0.93 | 0.74 |
| “Old” Blood | 0.99 | 0.05 | 1.00 | 15.66 | 0.93 | 0.73 |

**Table S6** DMRs identified by BSmooth (BS) or Metilene (ML) and further selected for subsequent DBS analysis.

| **DMR** | **Associated genes** | **Δ DNA methylation (old-young, WGBS)** |
| --- | --- | --- |
| BS21 | *MIR1471, NPPC* | -0.43 |
| BS74 | *EPO, EPHB4, ZAN* | -0.37 |
| BS88 | *LMX1B, ZBTB43* | -0.39 |
| BS134 | *SOX8, SSTR5* | -0.47 |
| ML15 | *LMNB2, TIMM13* | -0.75 |
| BS22 | *GBX2,* *ASB18* | -0.50 |
| BS71 | *SKAP2, HOXA1* | 0.39 |
| BS174 | *SHOX, CRLF2* | 0.32 |
| BS130 | *PWAR, SNORD115-1* | 0.42 |
| BS46 | *TRPC7, SPOCK1* | 0.38 |
| MS12 | *BCL6B,* *SLC16A13* | 0.52 |

Table7 Sequence of oligonucleotides used for relative telomere length analysis and Deep Bisulfite Sequencing

| Target | Primer sequence (5’-3’) | PCR conditions |
| --- | --- | --- |
| Telomere repeats | F: CGGTTTGTTTGGGTTTGGGTTTGGGTTTGGGTTTGGGTT  R: GGCTTGCCTTACCCTTACCCTTACCCTTACCCTTACCCT | See text |
| HBG | F: TGTGCTGGCCCATCACTTTG  R: ACCAGCCACCACTTTCTGATAGG | See text |
| BS21 *(MIR1471, NPPC)* | F: cttgcttcctggcacgag-GGTTTTTTAAATTAGAGTTAGTTGTGTTT  R: caggaaacagctatgac-CTAAAATAATTTTTATCCCATTCCC | 95ºC, 15’; 50x(96ºC, 15’’; 56ºC, 20’’; 72ºC, 30’’); 72ºC, 10’ |
| BS74 *(EPO, EPHB4, ZAN)* | F: cttgcttcctggcacgag-GATAGTGTTTTTTTGTTTTTAATTTATATA  R: caggaaacagctatgac-AATAATTTATCCAAAACCCTATACC | 95ºC, 15’; 50x(96ºC, 15’’; 54ºC, 20’’; 72ºC, 30’’); 72ºC, 10’ |
| BS88 *(LMX1B, ZBTB43)* | F: cttgcttcctggcacgag-AAAATATATATGGTTTTTTATTGAAT  R: caggaaacagctatgac-CCCAAACTACTCCTACCCCTACTAC | 95ºC, 15’; 50x(96ºC, 15’’; 60ºC, 20’’; 72ºC, 30’’); 72ºC, 10’ |
| BS134 *(SOX8, SSTR5)* | F: cttgcttcctggcacgag-TATATGGAAATGGAGTTTGTAGTGTG  R: caggaaacagctatgac-CTTCTAAAAAAACCCCAAAAAACTT | 95ºC, 15’; 50x(96ºC, 15’’; 56ºC, 20’’; 72ºC, 30’’); 72ºC, 10’ |
| ML15 *(LMNB2, TIMM13)* | F: cttgcttcctggcacgag –TGGGTAGTTAGAATGTAGGTTTGGT  R: caggaaacagctatgac-CACCATTTAATCAAATTAAAAACCC | 95ºC, 15’; 50x(96ºC, 15’’; 56ºC, 20’’; 72ºC, 30’’); 72ºC, 10’ |
| BS22 *(GBX2,* *ASB18)* | F: cttgcttcctggcacgag-AGTATAATTTTGGTTTGTTTTTTTG  R: caggaaacagctatgac-CTTCCTACCATATAAAATCCTACCC | 95ºC, 15’; 50x(96ºC, 15’’; 56ºC, 20’’; 72ºC, 30’’); 72ºC, 10’ |
| BS71 *(SKAP2, HOXA1)* | F: cttgcttcctggcacgag-ATATATAATTTTTATGGGTTAAAGTTAGAA  R: caggaaacagctatgac-AATTAACCAACAACTCCTAATAAATAC | 95ºC, 15’; 50x(96ºC, 15’’; 56ºC, 20’’; 72ºC, 30’’); 72ºC, 10’ |
| BS174 *(SHOX, CRLF2)* | F: cttgcttcctggcacgag-TGATGGGTAAATTAGGATTGT  R: caggaaacagctatgac-AAACTCCCATTCCAACAAAAA | 95ºC, 15’; 50x(96ºC, 15’’; 56ºC, 20’’; 72ºC, 30’’); 72ºC, 10’ |
| BS130 *(PWAR, SNORD115-1)* | F: cttgcttcctggcacgag-TAAATAATTTAAAAGTAAAATGATGTAAGA  R: caggaaacagctatgac-AAAAAATTACAACCTTAACCTAAAC | 95ºC, 15’; 50x(96ºC, 15’’; 53.5ºC, 20’’; 72ºC, 30’’); 72ºC, 10’ |
| BS46 *(TRPC7, SPOCK1)* | F: cttgcttcctggcacgag-TTATTAGAAGGAAGAAATTTTGAA  R: caggaaacagctatgac-AAAAAATAATAATAAACCCAAAAACCTAT | 95ºC, 15’; 50x(96ºC, 15’’; 56ºC, 20’’; 72ºC, 30’’); 72ºC, 10’ |
| MS12 *(BCL6B,* *SLC16A13)* | F: cttgcttcctggcacgag-TTTTTATGTTTTTTGTTTTTAGGGG  R: caggaaacagctatgac-CCCACCTCTTACAAATTTACCTTAA | 95ºC, 15’; 50x(96ºC, 15’’; 56ºC, 20’’; 72ºC, 30’’); 72ºC, 10’ |


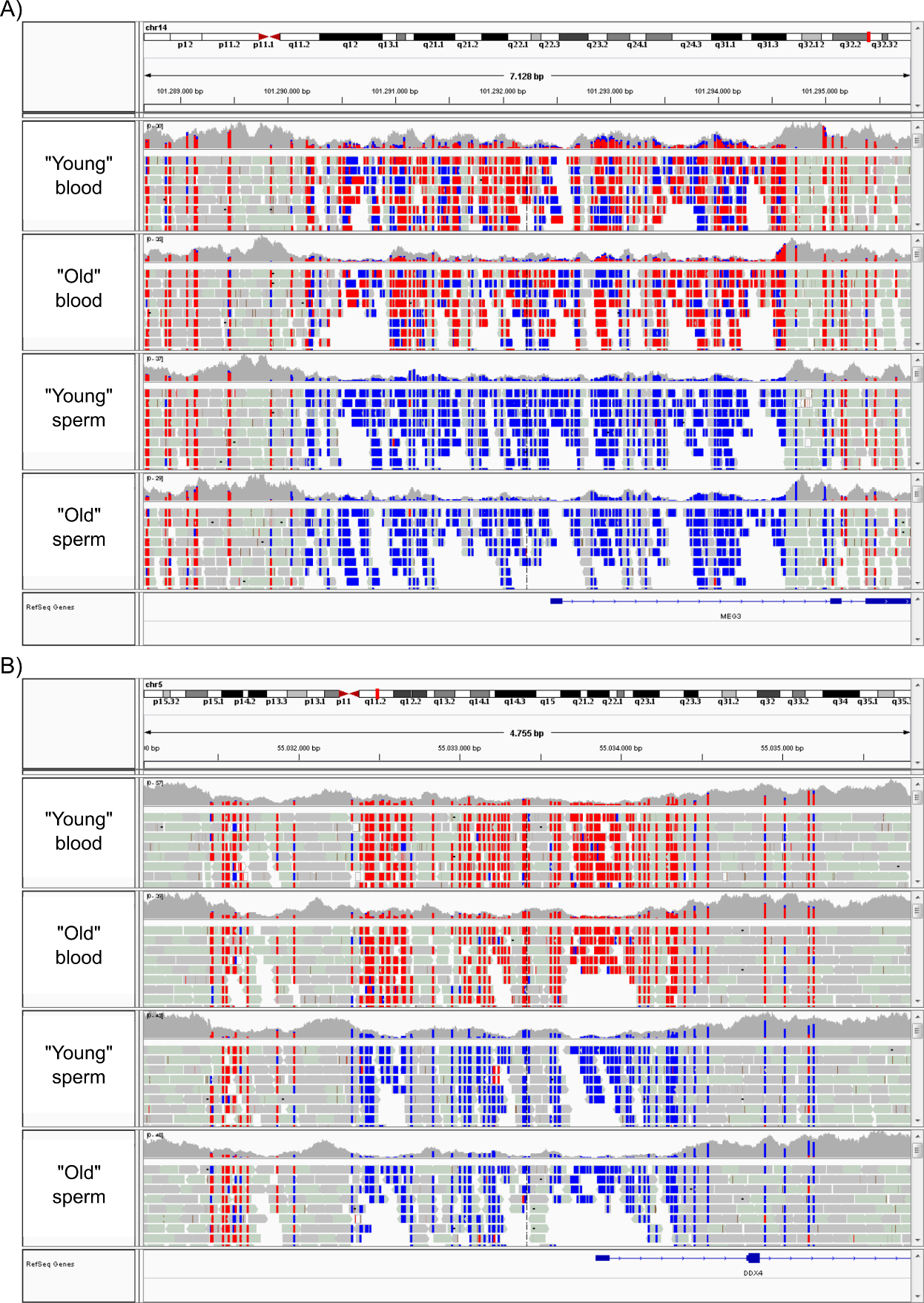


**Figure S1** IGV browser screenshots showing DNA methylation patterns of (A) MEG3 and (B) DDX4/VASA genes in the analysed samples. As a check for the purity of the samples and the quality of the WGBS data, we inspected imprinted genes such as MEG3 as well as the male germ cell specific DDX4 gene. There is an equal proportion of methylated and unmethylated MEG3 reads derived from blood DNA, but only unmethylated reads derived from sperm DNA, as expected for a maternally methylated imprinted gene. The same is true for other imprinted regions. As expected for DDX4, it is almost completely methylated in blood and completely unmethylated in sperm regardless of age. Red, methylated CpG; blue, unmethylated CpG.


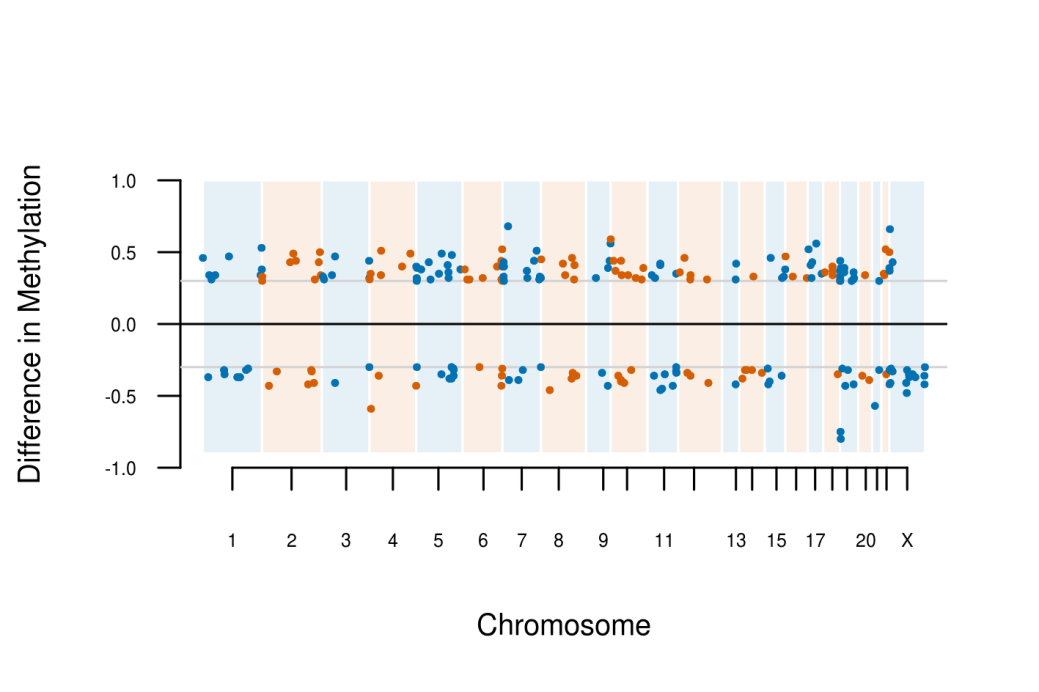


**Figure S2** Chromosomal distribution of sperm DMRs. Chromosomal localization of either hyper- or hypomethylated DMRs, plotted based on Stephen Turner's "qqman" R package [1]. DMRs obtained using "bsseq" and "metilene" with a methylation difference (y-axis) ranging from 0% (0) to 100% (1/-1) with a minimum methylation difference of 30% (0.3/-0.3) between groups. Chromosomes 1-22 + "X" on the x-axis. No DMRs could be observed on the Y Chromosome, which is not shown. Contrastingly coloured columns and dots refer to the different chromosomes.

**
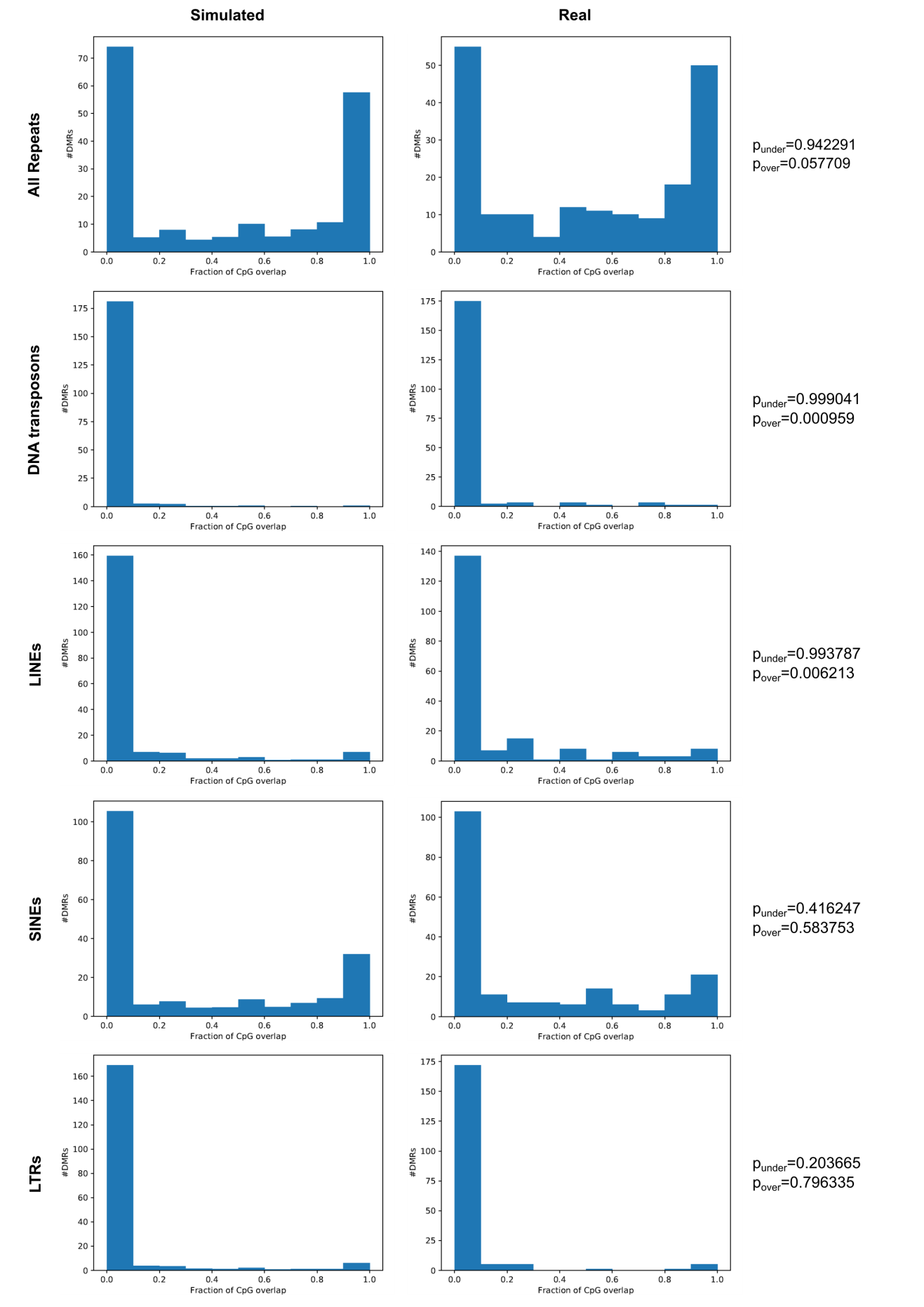
**

**Figure S3** Overlap of DMRs with repeat elements in the genome (all repeats, DNA transposons, LINEs – long interspersed nuclear elements, SINEs – short interspersed nuclear elements, and LTRs – long terminal repeats). Empirical p values for under- and over-representation in the dataset, compared to 1 million simulated datasets, were estimated. Only LINEs and DNA transposons were significantly over-represented in our dataset.


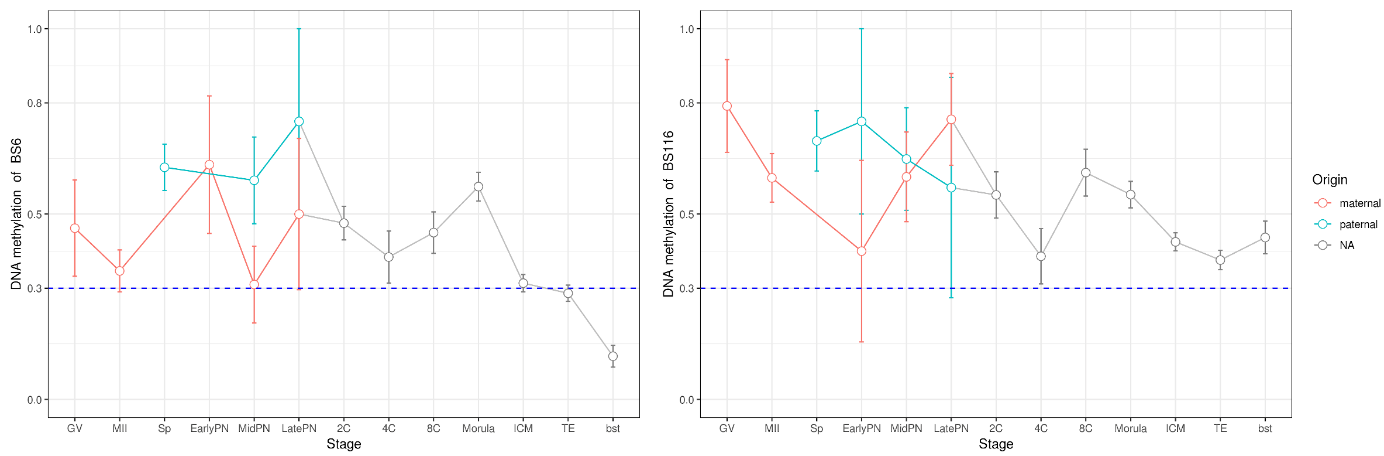


**Figure S4** Examples of DMRs that have the potential to escape genome-wide demethylation. Average methylation of DMR BS6 and BS116 is plotted in germinal vesicle (GV) and metaphase II (MII) oocytes, sperm (Sp), paternal (blue) and maternal (red) genomes in early (EarlyPN), mid (MidPN), and late (latePN) pronuclear stages, in 2-cell (2C), 4-cell (4C), 8-cell (8C) stage embryos, inner cell mass (ICM), trophectoderm (TE), and blastocyst (bst) stage embryos. A blue dashed line marks the 30% methylation threshold.

**
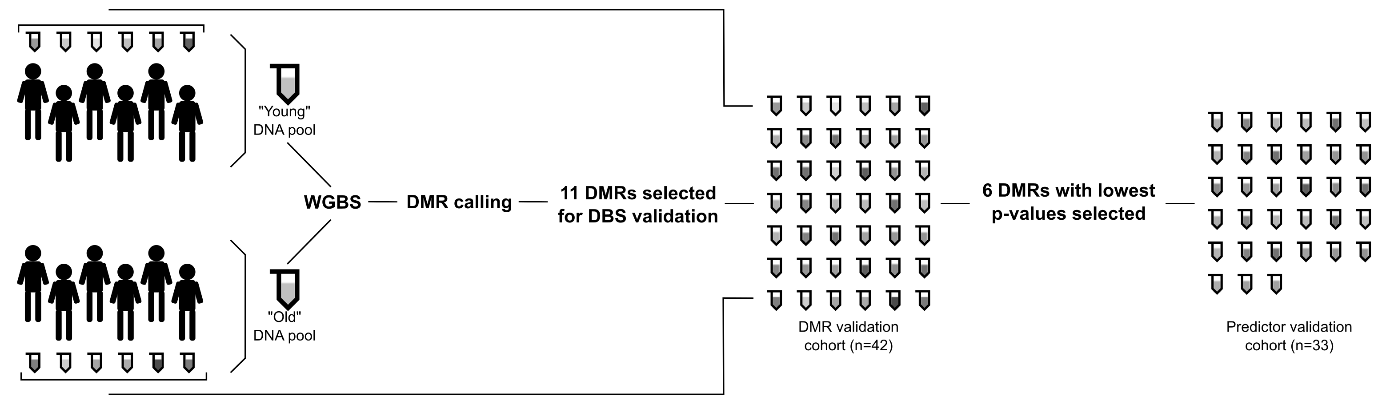
**

**Figure S5** Flowchart of semen samples used for DNA methylation analyses.
