# Supplementary material for: A germ cell‐specific ageing pattern in otherwise healthy men
Source: Aging Cell. 2020 Sep 20;19(10):e13242. doi: 10.1111/acel.13242 (PMC7576283; doi:10.1111/acel.13242)
Supplement: Supplementary file 4 [file ACEL-19-e13242-s004.docx]

**Supplementary File 3 – Experimental Procedures**

**Participants**

Participants were recruited through advertisements in local newspapers, internet, and hospitals. The recruitment and clinical evaluation/selection of the participants took place between October of 2014 and April of 2016. Prospective participants (above the age of 18) were first asked to fill an online questionnaire. Exclusion criteria in the questionnaire included smoking within the previous year, illegal drug consumption, regular use of medication (except for the treatment of mild hypertension, hypothyroidism, and dyslipoproteinemia), hospitalization within the previous month, current or former cancer/cancer treatment, severe chronic renal failure, chronic viral infection, urogenital malformations and surgeries, prior diagnosis or treatment for fertility impairment, chromosomal alterations, and participation in clinical trials within the previous year. 265 selected volunteers were invited to the outpatient clinic of the CeRA for clinical evaluation and sample collection. 65 volunteers dropped out for unwillingness or inability to make it to the appointment. 200 men underwent a thorough anthropometric, endocrine, and andrological evaluation, which was accompanied by several questionnaires addressing their wellbeing and sexual health. Two participants were further excluded due to the discovery of asymptomatic tumours during the clinical examination. 197 participants were ultimately included in the study.

Physical examination included measurement of body weight, height, blood pressure, pulse, and electrocardiogram. Blood samples were collected under fasting conditions and used for DNA isolation, hormone measurements, haematological analysis including metabolic parameters as blood lipids, HbA1c and blood glucose. Ultrasound of the thyroid, carotid arteries, kidneys, bladder, testicles, epididymis, prostate, and seminal vesicles (transrectal) in addition to palpation of breast, external genitalia, and digital rectal examination were performed. All collected data were stored in our in-house clinical database, Androbase (Tüttelmann, Luetjens, & Nieschlag, 2006).

Grouping was done in 10 year intervals, except for groups 1 (18-24 years) and 6 (> 65 years; range 65 - 82 years), the latter due to the difficulties in recruiting sufficient numbers of healthy men above 75 years of age (Table S1).

**Questionnaires**

General wellbeing including personal medical history, life time smoking habits, socio-economic factors, and family medical history were analysed using four different questionnaires. Clinical symptoms of age-related hypogonadism were evaluated using the Aging Males Symptoms (AMS) rating scale (Heinemann et al., 2003). Erectile function was assessed with the international index of erectile function (IIEF) (Rhoden, Teloken, Sogari, & Souto, 2002). The evaluation of clinical symptoms caused by an enlargement of the prostate was measured by the International Prostate Symptom Score questionnaire (Barry et al., 1992). Quality of daily sleep was assessed by the Epworth sleepiness scale (Johns, 1991).

**Hormone measurements**

Hormonal parameters (LH, FSH, T, SHBG, prolactin, estradiol, PSA) were measured using a chemiluminescent microparticle immunoassay (Architect i1000; Abbott Diagnostics, Wiesbaden, Germany). DHT was measured using an in-house radio-immunoassay. All blood samples were obtained in a fasting state after 15 minutes of rest. Methods are validated quarterly against LC-MS/MS.

**Sperm analysis and preparation**

The volunteers were also asked to provide at least 2 semen samples in two separate visits, with an abstinence time of 2 -7 days. The semen samples were analysed in our andrological lab in accordance with the WHO criteria (World Health Organization & Research, 2010). A microbiological analysis of semen was performed and swim-up sperm was prepared (World Health Organization & Research, 2010; Leitão et al., 2020) and stored for further analyses. Absence of contaminating cells was verified by visual inspection under the microscope.

**DNA isolation**

Blood DNA was isolated using EDTA-blood using the FlexiGene DNA kit (Qiagen) according to the instructions supplied by the manufacturer and as previously described (Laurentino et al., 2015). Sperm DNA was isolated from swim-up sperm using MasterPure DNA purification kit (Epicentre Biotechnologies, Madison, WI, USA) and a modified protocol (Laurentino et al., 2015). DNA concentration was measured by spectrophotometry (NanoDrop ND-1000, Peqlab, Erlangen, Germany). DNA samples were stored at -20ºC until further use.

**Relative telomere length analysis**

Mean relative telomere length (rTL) was measured on blood and swim-up sperm DNA by quantitative PCR (qPCR) using a previously described method (Meyer, Salewsky, Buchmann, Steinhagen-Thiessen, & Demuth, 2016), modified from (Cawthon, 2002). Briefly, the cycle threshold (Ct) value for telomeric repeats (T) and a single-copy gene [S; haemoglobin subunit gamma (*HBG*)] were determined for each sample and the rTL was calculated as the ratio between T and S. A standard curve was generated using reference DNA ranging from 0.37 to 90 ng from 10 randomly selected subjects. Measurements for subjects and reference samples were carried out in triplicates. Amplification mixture contained 10 µl QuantiTect SYBR^®^ green PCR Master Mix (2x; Qiagen), 2 µl blood DNA (12,5 ng/μl), 1 µl primer either for telomeric repeats (forward 2 µM, reverse 18 µM; Eurofins MWG) or *HBG* (forward 6 µM, reverse 14 µM; Eurofins MWG; Table S6, 2 µl DTT (25 mM), and nuclease free water to a total volume of 20 µl.

**DNA Fragmentation Index (DFI) determination**

Measurement of DNA fragmentation was performed as described previously (Evenson, 2016). For the flow cytometer setup and calibration, a reference sample was used from a normal donor. Data were analysed using the FCS 3.0 software package (DeNovo software, Los Angeles, CA, USA). A DNA fragmentation index (DFI) for every detected sperm was calculated according to the formula: red/(red + green) fluorescence. DFI values were plotted in a histogram and sperm with denatured DNA (high DFI values) were those located to the right of the main, normal population.

**Library preparation and sequencing**

Sperm and blood DNA, respectively, from six “young” men (18-24 years) and six “old” men (61-71 years) were pooled at equimolar ratios. We used pooled DNA to minimize the effect of inter-individual DNA methylation differences (Cheung et al., 2017; Do et al., 2016; Wallner et al., 2016). For each of the 4 pools, two shotgun libraries were generated. Standard pre-bisulfite libraries were prepared from 2 μg of DNA according to the original Illumina protocol essentially as described by Rademacher et al. (2014). Tagmentation libraries were prepared from 20 ng of DNA using the Nextera transposase (Illumina, San Diego, CA, USA) and a modified protocol (Souren et al., 2019). The employment of these two library preparation strategies in parallel in allows homogenous coverage of the genome. Bisulfite conversion was performed with the EZ DNA Methylation-Gold Kit (Zymo Research, Irvine, CA, USA), followed by 10 and 12 cycles of PCR, respectively. For each sample, we sequenced two lanes of the Illumina library and one lane of the tagmentation library on an Illumina HiSeq 2500, again as described previously (Rademacher et al., 2014).

Whole genome bisulfite sequencing (WGBS) data was deposited at the European Nucleotide Archive under the accession number PRJEB28044.

**Read mapping and methylation calling**

Raw reads were aligned with bwa-meth (v0.2.0) (Pedersen, Eyring, De, Yang, & Schwartz, 2014) to the human reference genome (hs37d5) with default parameters. Aligned reads were sorted and indexed, duplicates were detected, and alignment metrics were extracted by sambamba (v.0.6.6) (Tarasov, Vilella, Cuppen, Nijman, & Prins, 2015). Methylation levels at CpG dinucleotides (with a minimum coverage of 5 reads) were called by an in-house script, which considers only CpGs located on the reference genome.

**DMR detection**

Differentially methylated regions (DMRs) were detected by Metilene (v.0.2-7) (Jühling et al., 2016) and by an in-house script based on Bsmooth (Hansen, Langmead, & Irizarry, 2012). We chose to use these two algoritms as they detect preferentially shorter and longer DMRs, respectively. We removed the smoothing and detected differentially methylated CpGs (DMCs) by t-test as previously described ((p<0.01; Hansen et al., 2012). The statistical parameters for BSmooth were applied as originally described (Hansen et al., 2012). Only CpG sites with a minimum of 5 reads in all datasets were included in the analysis. Adjacent differentially methylated cytosines (DMCs) are merged into a DMR if they are separated by not more than two non-DMCs. DMRs containing four DMCs and a minimum of 0.3 (i.e. 30%) average group methylation difference between these DMCs were selected.

**Overlap of DMRs with repeats**

Repeat tracks produced by RepeatMasker (Tarailo-Graovac & Chen, 2009) were retrieved by the UCSC Table Browser (Karolchik et al., 2004). Separated BED files for the four different types of repeats were generated and intersected with CpG positions. This results in four BED files, each containing CpGs located in the respective repeat regions. CpGs of DMRs located in repeat regions were then identified by intersecting the DMRs in BED format with each of the four files. All operations on BED files are performed by BEDTools (v2.25.0) (Quinlan & Hall, 2010).

We estimated the p-value for over- and underrepresented repeat overlaps by simulating 1 million datasets, consisting of 236 regions each and equal size distribution compared to our DMRs, while each region contains at least 4 CpGs. For each of our 236 DMRs and each region of the simulated datasets, the fraction of repeat-overlapping CpGs was detected. We then compared the mean fraction f for our DMRs and each of the 1 million random sets F'. The empirical p-value for overrepresentation is the fraction of random sets f' ∈ F' with f > f' (symmetrically, for underrepresentation). This was performed independently for each type of repeat.

**Detection of potential escapee regions**

For detection of regions potentially escaping the first wave of genome wide demethylation, publicly available WGBS data was used (GSE81233; Zhu et al., 2018). This dataset contains single cell methylome data from human sperm (n=21), germinal vesicle (GV; n=6) and meiosis II (MII; n=33) oocytes, and early (n=7), mid (n=15), and late (n=8) pronuclear zygotes. In brief, BED files were parsed and transformed into GRanges objects (Lawrence et al., 2013). Due to the low coverage of single cell WGBS data, no coverage threshold was set. Methylation data could be retrieved for 225 out of the 236 DMRs for all stages. We considered as potential escapee regions those that had persistent DNA methylation above 30% and a variation in DNA methylation below 30% until late pronucleus stage.

**Gene Ontology Analysis**

Annotation of the DMRs was performed by searching the nearest genes within 100 kb. The resulting gene list was submitted to DAVID (Huang, Sherman, & Lempicki, 2009) was used for gene ontology, using the whole human genome as backgroun. GSEA (Subramanian et al., 2005) was used for gene set enrichment analysis using the Molecular Signatures Database (MsigDB 3.0)(Liberzon et al., 2011). This tool calculates the overlap between a provided list of genes and MSigDB collections.

**Deep Bisulfite Sequencing (DBS)**

To select DMRs for validation, we excluded those which overlapped with repetitive regions which lead to difficulties in designing specific primer sets. From those, we selected DMRs showing the strongest differences in DNA methylation between young and old according to WGBS. We tested 19 primer sets,designed using MethPrimer (Li & Dahiya, 2002) (http://www.urogene.org/cgi-bin/methprimer/methprimer.cgi) and MethPrimer 2.0 (http://www.urogene.org/cgi-bin/methprimer2/MethPrimer.cgi). Eight primer sets did not perform according to our standards either at the PCR or the sequencing stage. Ultimately, eleven DMRs (Table S7) passed our internal selection and quality control criteria and were used for validation. Briefly, DMR-specific libraries were constructed by two rounds of PCR as previously described (Beygo, Ammerpohl, et al., 2013; Laurentino et al., 2015). Sample preparation and sequencing were performed as previously described (Beygo, Ammerpohl, et al., 2013) using the Roche/454 GS Junior system (Roche Diagnostics, Manheim, Germany) and yield of reads was increased by applying a special filter setting (Beygo, Citro, et al., 2013). A power analysis was performed to find the minimum number of samples to be analysed (p<0.05 and β<0.2, large effect size). Forty two samples (which included the individual 12 samples previously analysed as pools by WGBS) were analysed for the 11 DMRs (Figure S4).

Methylation analysis was performed using Amplikyzer (Rahmann et al., 2013), and average methylation for each amplicon and each CpG site, as well as methylation plots, were retrieved for each sample.

**Epigenetic age-predictor**

In order to develop a mathematical model for the prediction of age based on the DNA methylation of six DMRs, we let X be the set of Donors, D our six selected DMRs, mx(d) be the average methylation level (if available) for DMR d ∈ D and donor x ∈ X and a(x) its age.

We use the set Ti,j = {(mx(di), mx(dj), a(x)) for x ∈ X} to separately train a ridge regression (Hoerl & Kennard, 1970) predictor pi,j for each pair (di, dj) ∈ D2 with 0 <= i < j < |D|, while elements with at least one missing methylation value for x are removed from Ti,j.

Each pi,j is able to predict an age a'i,j(x) = pi,j(mx(di), mx(dj)) given average methylation of x for di and dj. Let then a'(x) be the average of all single predictions for x.

In a second step we use all pairs T'i,j = {(a'(x), a(x)) for x ∈ X} to train an additional simple linear regression model q. The final predicted age for x is is q(a'(x)).

The predictor was validated using a cohort of 33 randomly selected sperm DNA samples (Figure S4).

**Statistics**

For each analysed variable, normality of the distribution and homogeneity of variance were tested before determining the most adequate statistical test to use. Two-sided t-tests were used to compare between two groups. Pearson’s test was used to test correlations between age and normally distributed variables. Spearman’s rank correlation was used when the variables were not normally distributed or presented outliers. Adjustment for confounders was done by partial correlation analysis. All statistical analysis and graph plotting were performed using R 3.3.1 and suitable R packages.

**References to the Experimental Procedures**

Barry, M. J., Jr, F. J. F., O’Leary, M. P., Bruskewitz, R. C., Holtgrewe, H. L., Mebust, W. K., & Cockett, A. T. (1992). The American Urological Association symptom index for benign prostatic hyperplasia. The Measurement Committee of the American Urological Association. *The Journal of Urology*, *148*(5), 1549–1557; discussion 1564. https://doi.org/S0022-5347(17)36966-5 [pii]

Beygo, J., Ammerpohl, O., Gritzan, D., Heitmann, M., Rademacher, K., Richter, J., … Buiting, K. (2013). Deep bisulfite sequencing of aberrantly methylated Loci in a patient with multiple methylation defects. *PloS One*, *8*(10), e76953. https://doi.org/10.1371/journal.pone.0076953 [doi]

Beygo, J., Citro, V., Sparago, A., Crescenzo, A. D., Cerrato, F., Heitmann, M., … Riccio, A. (2013). The molecular function and clinical phenotype of partial deletions of the IGF2/H19 imprinting control region depends on the spatial arrangement of the remaining CTCF-binding sites. *Human Molecular Genetics*, *22*(3), 544–557. https://doi.org/10.1093/hmg/dds465 [doi]

Cawthon, R. M. (2002). Telomere measurement by quantitative PCR. *Nucleic Acids Research*, *30*(10), e47.

Cheung, W. A., Shao, X., Morin, A., Siroux, V., Kwan, T., Ge, B., … Grundberg, E. (2017). Functional variation in allelic methylomes underscores a strong genetic contribution and reveals novel epigenetic alterations in the human epigenome. *Genome Biology*, *18*(1), 50. https://doi.org/10.1186/s13059-017-1173-7

Do, C., Lang, C. F., Lin, J., Darbary, H., Krupska, I., Gaba, A., … Tycko, B. (2016). Mechanisms and Disease Associations of Haplotype-Dependent Allele-Specific DNA Methylation. *American Journal of Human Genetics*, *98*(5), 934–955. https://doi.org/10.1016/j.ajhg.2016.03.027

Evenson, D. P. (2016). The Sperm Chromatin Structure Assay (SCSA((R))) and other sperm DNA fragmentation tests for evaluation of sperm nuclear DNA integrity as related to fertility. *Animal Reproduction Science*, *169*, 56–75. https://doi.org/10.1016/j.anireprosci.2016.01.017 [doi]

Hansen, K. D., Langmead, B., & Irizarry, R. A. (2012). BSmooth: From whole genome bisulfite sequencing reads to differentially methylated regions. *Genome Biology*, *13*(10), R83-2012-13-10-r83. https://doi.org/10.1186/gb-2012-13-10-r83 [doi]

Heinemann, L. A., Saad, F., Zimmermann, T., Novak, A., Myon, E., Badia, X., … Giroudet, C. (2003). The Aging Males’ Symptoms (AMS) scale: Update and compilation of international versions. *Health and Quality of Life Outcomes*, *1*, 15.

Huang, D. W., Sherman, B. T., & Lempicki, R. A. (2009). Systematic and integrative analysis of large gene lists using DAVID bioinformatics resources. *Nature Protocols*, *4*(1), 44–57. https://doi.org/10.1038/nprot.2008.211

Johns, M. W. (1991). A new method for measuring daytime sleepiness: The Epworth sleepiness scale. *Sleep*, *14*(6), 540–545.

Jühling, F., Kretzmer, H., Bernhart, S. H., Otto, C., Stadler, P. F., & Hoffmann, S. (2016). Metilene: Fast and Sensitive Calling of Differentially Methylated Regions from Bisulfite Sequencing Data. *Genome Research*, *26*(2), 256–262. https://doi.org/10.1101/gr.196394.115 [doi]

Karolchik, D., Hinrichs, A. S., Furey, T. S., Roskin, K. M., Sugnet, C. W., Haussler, D., & Kent, W. J. (2004). The UCSC Table Browser data retrieval tool. *Nucleic Acids Research*, *32*(Database issue), D493-6. https://doi.org/10.1093/nar/gkh103 [doi]

Laurentino, S., Beygo, J., Nordhoff, V., Kliesch, S., Wistuba, J., Borgmann, J., … Gromoll, J. (2015). Epigenetic germline mosaicism in infertile men. *Human Molecular Genetics*, *24*(5), 1295–1304. https://doi.org/10.1093/hmg/ddu540 [doi]

Lawrence, M., Huber, W., Pages, H., Aboyoun, P., Carlson, M., Gentleman, R., … Carey, V. J. (2013). Software for computing and annotating genomic ranges. *PLoS Computational Biology*, *9*(8), e1003118. https://doi.org/10.1371/journal.pcbi.1003118 [doi]

Leitão, E., Di Persio, S., Laurentino, S., Wöste, M., Dugas, M., Kliesch, S., … Horsthemke, B. (2020). The sperm epigenome does not display recurrent epimutations in patients with severely impaired spermatogenesis. *Clinical Epigenetics*, *12*(1), 61. https://doi.org/10.1186/s13148-020-00854-0

Li, L. C., & Dahiya, R. (2002). MethPrimer: Designing primers for methylation PCRs. *Bioinformatics (Oxford, England)*, *18*(11), 1427–1431.

Liberzon, A., Subramanian, A., Pinchback, R., Thorvaldsdottir, H., Tamayo, P., & Mesirov, J. P. (2011). Molecular signatures database (MSigDB) 3.0. *Bioinformatics*, *27*(12), 1739–1740. https://doi.org/10.1093/bioinformatics/btr260

Meyer, A., Salewsky, B., Buchmann, N., Steinhagen-Thiessen, E., & Demuth, I. (2016). Relative Leukocyte Telomere Length, Hematological Parameters and Anemia—Data from the Berlin Aging Study II (BASE-II). *Gerontology*, *62*(3), 330–336. https://doi.org/10.1159/000430950 [doi]

Pedersen, B. S., Eyring, K., De, S., Yang, I. V., & Schwartz, D. A. (2014). Fast and accurate alignment of long bisulfite-seq reads. *arXiv*.

Quinlan, A. R., & Hall, I. M. (2010). BEDTools: A flexible suite of utilities for comparing genomic features. *Bioinformatics (Oxford, England)*, *26*(6), 841–842. https://doi.org/10.1093/bioinformatics/btq033 [doi]

Rademacher, K., Schroder, C., Kanber, D., Klein-Hitpass, L., Wallner, S., Zeschnigk, M., & Horsthemke, B. (2014). Evolutionary Origin and Methylation Status of Human Intronic CpG Islands that Are Not Present in Mouse. *Genome Biology and Evolution*, *6*(7), 1579–1588. https://doi.org/10.1093/gbe/evu125 [doi]

Rahmann, S., Beygo, J., Kanber, D., Martin, M., Horsthemke, B., & Buiting, K. (2013). Amplikyzer: Automated methylation analysis of amplicons from bisulfite flowgram sequencing. *PeerJ Preprints*. https://doi.org/10.7287/peerj.preprints.122v2

Rhoden, E. L., Teloken, C., Sogari, P. R., & Souto, C. A. V. (2002). The use of the simplified International Index of Erectile Function (IIEF-5) as a diagnostic tool to study the prevalence of erectile dysfunction. *International Journal of Impotence Research*, *14*(4), 245–250. https://doi.org/10.1038/sj.ijir.3900859 [doi]

Subramanian, A., Tamayo, P., Mootha, V. K., Mukherjee, S., Ebert, B. L., Gillette, M. A., … Mesirov, J. P. (2005). Gene set enrichment analysis: A knowledge-based approach for interpreting genome-wide expression profiles. *Proceedings of the National Academy of Sciences of the United States of America*, *102*(43), 15545–15550. https://doi.org/0506580102 [pii]

Tarailo-Graovac, M., & Chen, N. (2009). Using RepeatMasker to identify repetitive elements in genomic sequences. *Current Protocols in Bioinformatics*, *Chapter 4*, Unit 4.10. https://doi.org/10.1002/0471250953.bi0410s25 [doi]

Tarasov, A., Vilella, A. J., Cuppen, E., Nijman, I. J., & Prins, P. (2015). Sambamba: Fast processing of NGS alignment formats. *Bioinformatics (Oxford, England)*, *31*(12), 2032–2034. https://doi.org/10.1093/bioinformatics/btv098 [doi]

Tüttelmann, F., Luetjens, C. M., & Nieschlag, E. (2006). Optimising workflow in andrology: A new electronic patient record and database. *Asian Journal of Andrology*, *8*(2), 235–241. https://doi.org/10.1111/j.1745-7262.2006.00131.x

Wallner, S., Schroder, C., Leitao, E., Berulava, T., Haak, C., Beisser, D., … Horsthemke, B. (2016). Epigenetic dynamics of monocyte-to-macrophage differentiation. *Epigenetics & Chromatin*, *9*, 33. https://doi.org/10.1186/s13072-016-0079-z

World Health Organization, D. of R. H., & Research. (2010). *WHO laboratory manual for the examination and processing of human semen—Fifth edition*.

Zhu, P., Guo, H., Ren, Y., Hou, Y., Dong, J., Li, R., … Tang, F. (2018). Single-cell DNA methylome sequencing of human preimplantation embryos. *Nature Genetics*, *50*(1), 12–19. https://doi.org/10.1038/s41588-017-0007-6 [doi]
